# 3D Bioprinting for Custom Bone Grafts in Orthopaedic Trauma: Current Advances and Clinical Translation

**DOI:** 10.7759/cureus.96594

**Published:** 2025-11-11

**Authors:** Gaurav Jha, Gagandeep Mahi, Surya Malasani, Mohamed Onsa, Ahmed Barakat

**Affiliations:** 1 Trauma and Orthopaedics, Leicester Royal Infirmary, Leicester, GBR; 2 Trauma and Orthopaedics, University Hospitals of Leicester NHS Trust, Leicester, GBR

**Keywords:** 3d bioprinting, bone tissue engineering, custom bone grafts, orthopaedic trauma, segmental bone defects, traumatic bone loss

## Abstract

Traumatic bone loss in orthopaedic trauma presents significant clinical challenges, particularly arising from high-energy injuries, open fractures with segmental defects, and blast trauma, where bone loss exceeds the critical size for spontaneous healing. Traditional management strategies, including autografts, allografts, and the Masquelet-induced membrane technique, are associated with limitations such as donor site morbidity, prolonged treatment duration, multiple surgical procedures, and suboptimal outcomes. Three-dimensional (3D) bioprinting technology has emerged as a promising solution for fabricating patient-specific bone grafts tailored to individual defect geometry and mechanical requirements.

This narrative review examines current advances in 3D bioprinting specifically for orthopaedic trauma applications, focusing on technologies relevant to long bone reconstruction, load-bearing requirements, and complex fracture management. We analyze various bioprinting modalities, including extrusion-based and stereolithography techniques, alongside biomaterial developments in bioceramics, biodegradable polymers, and mechanically competent composite scaffolds. Clinical applications in femoral and tibial segmental defects, periarticular fractures, and combat-related extremity injuries are reviewed, with emphasis on preclinical large animal models and early clinical experiences. Critical challenges specific to orthopaedic trauma, including mechanical loading demands, infection risk in contaminated wounds, and integration with existing fixation systems, are discussed.

This review examines emerging innovations, such as in situ bioprinting for intraoperative graft fabrication and personalized approaches incorporating patient-derived cells and growth factors. As bioprinting technology continues to mature, integration into orthopaedic trauma protocols promises to reduce treatment complexity, accelerate healing, and improve functional outcomes for patients with devastating skeletal injuries requiring advanced reconstructive solutions.

## Introduction and background

Excessive bone loss resulting from orthopaedic trauma constitutes a significant challenge in musculoskeletal surgery. Road traffic accidents, falls from height, gunshot wounds, and blast injuries often result in high-energy trauma causing complex bone loss patterns, including segmental defects, comminution with fragment loss, and open fractures with significant contamination [1]. Bone defects of a size larger than critical, i.e., larger than two to 2.5 times the diameter of the bone involved, hardly heal spontaneously. Surgical reconstruction must be performed in such cases to preserve bone continuity and function [2,3].

Autologous bone grafting, which involves the transplantation of vascularized or non-vascularized bone from the iliac crest, fibula, or other donor sites, remains the gold standard for the treatment of large traumatic bone defects [4]. Whereas autografts contain osteogenic cells, osteoinductive growth factors, and an osteoconductive matrix, they are limited by donor site morbidity (8% to 39% of the patients), reduced graft volume, less than ideal integration, risk of infection, and insufficient mechanical strength [5,6]. Other described methods, such as the Masquelet-induced membrane technique [6], encompass multiple surgical stages over several weeks, and distraction osteogenesis using external fixators requires lengthy treatment stages and high levels of patient compliance [7,8]. Allografts and synthetic bone substitutes for grafting address the issue of graft supply but have drawbacks in mechanical strength, host bone incorporation, infection risk, and immune response. 

These historical constraints have driven the development of tissue engineering strategies aimed at merging biological performance and mechanical integrity and minimizing donor site morbidity [9]. Three-dimensional (3D) bioprinting as a cutting-edge technology for the production of patient-specific bone grafts has addressed the anatomical and biomechanical requirements of trauma surgery [10]. Unlike conventional bone substitutes, bioprinted scaffolds are printed from CT image data of the defect to ensure precise anatomical matching even in irregular or segmental defects. Moreover, bioprinting enables scaffold stiffness to be tailored to match local bone density, incorporation of internal fixation devices, pore structure design for vascularization, and addition of antimicrobial or osteogenic agents for healing contaminated or complex wounds [11,12]. 

This narrative review integrates current information on 3D bioprinting technologies dedicated to orthopaedic trauma applications, with emphasis on clinical contexts in fracture surgery and limb reconstruction. We cover bioprinting strategies to develop mechanically stable constructs for load-bearing purposes, biomaterials progress in coping with the demanding nature of traumatic bone reconstruction, and clinical translation advances in long bone segmental defects. 

## Review

Bioprinting technologies for orthopaedic applications

Extrusion-Based Bioprinting for Load-Bearing Scaffolds

Extrusion-based bioprinting stands out as the most clinically relevant technology for orthopaedic trauma, primarily because it can process highly viscous materials, produce clinically relevant-sized constructs, and achieve mechanical properties appropriate for load-bearing applications [13]. The process dispenses bioink ranging from thermoplastic polymers such as polycaprolactone to bioceramics like calcium phosphate and hydroxyapatite, or even composite blends, through a nozzle using controlled pneumatic or mechanical pressure. In this review, bioink refers to printable formulations containing biologically active components such as ceramics, growth factors, or cells, whereas printable materials denote acellular polymers and composites used primarily for structural support. This process is based on computer-aided design models generated from patient CT scans, enabling the creation of patient-specific scaffolds that fit anatomical requirements [14].

For long bone defects in the femur and tibia, extrusion bioprinting can produce anatomically contoured or cylindrical scaffolds ranging from 2 cm to 15 cm in length, with diameters suitable for medullary canal filling or cortical bone replacement. A printing resolution of 100 to 500 µm enables porous architectures that balance strength and space for bone ingrowth [15]. Parameters such as strut thickness, pore size, and pore geometry are precisely controlled to optimize stress distribution and biological integration, guided by finite element analysis of patient-specific loading conditions [9].

Advanced extrusion systems utilize multiple print heads to deposit different materials in defined spatial patterns, which enables the fabrication of heterogeneous constructs that closely replicate native bone architecture [16]. For instance, dense outer shells that mimic cortical bone can be produced using mechanically robust polymers or polymer-ceramic composites. In contrast, porous inner cores that replicate trabecular bone architecture can incorporate more degradable materials and higher concentrations of bioactive factors. This biomimetic strategy addresses the dual challenge of providing initial mechanical support for early weight-bearing and facilitating biological remodeling over time [17].

Stereolithography and Light-Based Printing Technologies

Stereolithography and digital light processing (DLP) are advanced 3D printing technologies that utilize photopolymerization of liquid resins to achieve high-resolution and superior surface quality scaffolds, outperforming conventional extrusion-based methods [18]. Historically, their use in biomedical applications was limited by a lack of biocompatible, mechanically robust photosensitive resins. However, recent advancements such as the integration of ceramic and mineral-incorporated photopolymers have significantly expanded their utility in orthopaedic trauma. These technologies excel in producing complex geometries needed for periarticular fractures and metaphyseal defects, where irregular bone loss patterns require precise contouring [19,20]. 

The light-based bioprinting techniques routinely achieve resolution of 25 μm to 100 μm, facilitating reproduction of fine anatomical microarchitecture, including trabecular patterns, cortical microporosity, and subchondral bone topography [21]. At present, light-based bioprinting is most applicable to periarticular and metaphyseal reconstructions, such as tibial plateau and distal radius defects, where restoring subchondral architecture and supporting fixation plates are critical. Complete diaphyseal replacement remains a longer-term goal pending advances in material strength and vascularization. Such fidelity is especially beneficial in intra-articular fracture management, where defects are irregular and maintaining articular congruency is critical. The smooth surface finishes produced by stereolithography minimize stress risers that could initiate crack propagation under cyclic loading conditions, an important consideration for trauma applications where implants must withstand physiological forces during healing.

Furthermore, recent innovations in ceramic stereolithography have produced scaffolds with compressive strengths approximating those of native trabecular bone, thus supporting early-stage metaphyseal load-bearing [19]. Post-printing sintering enhances the mechanical properties and enables osteoconductive bonding to host bone via calcium phosphate surface chemistry, further improving integration and long-term stability.

In Situ Bioprinting for Intraoperative Graft Fabrication

In situ bioprinting represents a significant shift in trauma surgery practice, especially where defect geometry may be difficult to predict preoperatively due to debridement requirements, complex fracture patterns, or soft tissue damage [22]. In situ bioprinting, currently demonstrated mainly in preclinical and benchtop studies, enables direct deposition of printable materials into bone defects using handheld or robot-assisted devices. Limited early human feasibility reports exist, but this technique is not yet in routine clinical use [23]. Clinical use to date has been limited to isolated feasibility reports rather than routine operating room application. This approach eliminates dimensional mismatches between prefabricated implants and actual defect geometry, which often occur when CT imaging performed days or weeks before surgery does not reflect the final defect configuration after debridement. Recent preclinical studies have demonstrated that this approach can regenerate bone more effectively than standard off-the-shelf scaffolds or conventional bone substitutes. For example, researchers have used laser-assisted bioprinting to deliver mesenchymal stromal cells in collagen or nano-hydroxyapatite matrices directly onto mouse skull defects, and the results point toward faster, real-time, and more reliable bone repair [22].

Integration of in situ bioprinting with existing trauma workflows requires development of portable, sterilizable devices compatible with operating room environments and surgical draping [15]. The bioinks must demonstrate shelf stability for storage in hospital pharmacies or operating rooms, with reconstitution or activation processes simple enough for intraoperative use.

Biomaterials for load-bearing trauma applications

Mechanically Competent Polymer-Ceramic Composites

The mechanical requirements of load-bearing bone reconstruction in trauma patients exceed those required for non-load-bearing applications. Materials used in these scenarios must exhibit higher compressive strength, flexural rigidity, and fatigue resistance. Native cortical bone exhibits compressive strength of 130 MPa to 180 MPa and an elastic modulus of 15 MPa to 20 GPa, while trabecular bone demonstrates compressive strength of 2 MPa to 12 MPa and an elastic modulus of 0.1 GPa to 5 GPa. For 3D printed bone scaffolds to succeed in physiological conditions, their mechanical properties should approximate these native values to avoid failure [24, 5]. Polymer-ceramic composite bioinks have emerged as the most promising material class for these demanding applications. These composites have the ability to combine the processability and toughness of polymers with the stiffness and bioactivity of ceramics [17,26].

Polycaprolactone-hydroxyapatite composites are among the most extensively investigated formulations for orthopaedic bioprinting, with ceramic content typically ranging from 10% to 40% by weight [27]. Increasing hydroxyapatite content enhances compressive strength, elastic modulus, and osteoconductivity but reduces printability and can compromise fracture toughness [28]. Optimization studies suggest that incorporating compositions of 20% to 30% hydroxyapatite achieves a favorable balance between mechanical performance, printability, and biological integration, resulting in compressive strength values (15 MPa to 40 MPa) suitable for trabecular bone replacement and metaphyseal applications [29].

Recent advancements focus on the inclusion of nano-sized ceramic particles, which provide a larger surface area for cell interactions and improved dispersion within polymer matrices compared to traditional micro-sized particles. Nano-hydroxyapatite and nano-tricalcium phosphate composites demonstrate enhanced mechanical properties due to better interfacial bonding between ceramic and polymer phases. Additionally, nanoscale surface roughness resulting from particle incorporation promotes osteoblast adhesion and differentiation through mechanotransduction pathways, enhancing biological integration [14]. 

Bioceramic Scaffolds and Surface Modifications

Bioceramic materials such as hydroxyapatite, tricalcium phosphate, calcium phosphate, and bioactive glasses have excellent biocompatibility and biological activity with stable physical and chemical properties. These attributes have contributed to their extensive application in the field of regenerative medicine, particularly for bone defect repair [30]. These materials serve a dual function: they can effectively provide mechanical support and actively promote osteogenic differentiation of bone marrow mesenchymal stem cells, facilitating both in vitro and in vivo bone formation. For trauma applications where compromised soft tissues, infection, or patient comorbidities may delay healing, slow degradation is advantageous compared to faster-degrading polymers like polylactic-co-glycolic acid [10].

However, simple bioceramic powder cannot be used directly to repair bone defects of a certain size due to its rapid degradation and tendency to disperse. Therefore, bioceramics are fabricated into porous 3D scaffolds, which enable effective nutrient exchange and support the regeneration and repair of large-scale bone defects. Conventional scaffold preparation techniques, such as the pore-forming agent method, freeze-drying method, and foaming method, can achieve high porosity, but these techniques are difficult to produce scaffolds that accurately conform to complex defect geometries [30]. Incorporation of block copolymers such as polycaprolactone and polyethylene glycol can further enhance hydrophilicity and cell infiltration while modestly accelerating degradation [17]. 

Surface modification technologies, including coatings, can improve the surface performance of 3D-printed bioceramic scaffolds. Modification of the scaffold surface has significant benefits by greatly improving the adhesion and proliferation of cells on the scaffold surface. Additionally, functional surface treatments can strengthen the scaffold’s mechanical properties or introduce specialized features, such as antibacterial or photothermal effects, thereby expanding the versatility of traditional 3D-printed bioceramic scaffolds [20].

Growth Factor Incorporation for Enhanced Regeneration

Bone morphogenetic proteins (BMPs), particularly BMP-2 and BMP-7, are recognized as potent osteoinductive agents that have been successfully incorporated into bioprinted scaffolds for trauma applications. When BMPs are incorporated into scaffold matrices and released locally at the site of injury, they significantly enhance bone regeneration, even in complex clinical situations such as atrophic nonunions, infected bone defects, or critical-sized segmental defects where natural healing is improbable [31]. The principal mechanism of BMPs involves the recruitment of mesenchymal stem cells to the defect site, guiding their differentiation into osteogenic lineages, with BMP-2 being particularly effective in driving osteoblastic differentiation [9].

Bioprinting technology enables sophisticated spatiotemporal control of growth factor delivery through multiple mechanisms [17]. Growth factors can be physically entrapped within hydrogel components of composite bioinks, adsorbed onto ceramic particles, or encapsulated within biodegradable microspheres or nanoparticles that provide sustained release. Sequential delivery of multiple growth factors, like vascular endothelial growth factor (VEGF), is often introduced early to promote angiogenesis, followed by BMP-2 to stimulate osteogenesis. Such delivery can be regulated through the differential degradation of carrier materials or by spatial patterning during the bioprinting process [2]. Notably, dual delivery of BMP-2 and VEGF using gelatin or alginate scaffolds has been shown to enhance vascularization and subsequent bone formation, with the specific ratio of BMP-2 to VEGF influencing the observed synergistic effects [2,32]. While BMP-2 enhances osteogenesis, its therapeutic window is narrow, and excessive local concentrations have been associated with adverse inflammatory and ectopic bone formation in clinical contexts.

For contaminated trauma wounds at high infection risk, antimicrobial agents, including antibiotics, silver nanoparticles, or antimicrobial peptides, can be co-delivered with osteogenic factors within the bioprinted scaffolds [33,34]. Local antibiotic delivery from bioprinted scaffolds achieves tissue concentrations far exceeding systemic therapy while minimizing systemic toxicity, an important consideration for managing open fractures with extensive soft tissue injury and contamination. High local antibiotic concentrations may induce osteoblast toxicity, while subtherapeutic levels during the later release phase can promote bacterial resistance. Moreover, most existing evidence supporting antibiotic-loaded bioprinted scaffolds is derived from controlled laboratory or animal models rather than contaminated trauma wounds, limiting direct clinical applicability [23,30].

Clinical applications in orthopaedic trauma

Segmental Bone Defects in Long Bones

Segmental bone defects in the femur and tibia resulting from high-energy trauma, gunshot wounds, or debridement of infected nonunions remain the greatest challenges in orthopaedic trauma surgery. Traditional reconstruction with the Masquelet technique requires staged procedures over six to eight weeks and necessitates non-weight bearing for a prolonged duration. In large defects exceeding 5 cm, nonunion rates can reach 10% to 25%, underscoring the limitation of current options. Bioprinted bone grafts offer potential for single-stage reconstruction, reducing surgical burden and accelerating rehabilitation [6,8].

Preclinical studies in large animal models with critical-sized femoral defects of 3 cm to 5 cm have demonstrated comparable or superior bone regeneration with bioprinted polymer-ceramic composite scaffolds compared to autograft controls [35]. Notably, Pobloth et al. reported that mechanobiologically optimized 3D titanium-mesh scaffolds promoted bone healing in ovine segmental defects, with radiographic and mechanical assessments indicating restoration approaching the strength of intact bone [36]. Notably, animals bearing bioprinted scaffolds achieved earlier weight-bearing compared to autograft groups, suggesting adequate initial mechanical competence.

The largest published preclinical experience involves ovine and caprine models, which approximate human long bone dimensions and loading conditions more closely than rodent models [35,36]. In these studies, anatomically contoured bioprinted scaffolds, secured with intramedullary nails or locking plates, facilitated bridging of segmental defects. Over six to nine months, bone formation initiated at the scaffold periphery and advanced centrally. Histological analysis showed scaffold degradation coupled with bone remodeling, eventually leading to restoration of cortical continuity and reconstitution of the medullary canal [35].

Early clinical case reports have described the successful application of bioprinted scaffolds for tibial segmental defects in humans, though published experience remains limited [23]. Preliminary results suggest clinical feasibility, with patients returning to ambulation and work, though larger series with longer follow-up are needed to establish safety, efficacy, and optimal indications for this technology.

Metaphyseal and Periarticular Fractures

Complex intra-articular fractures, such as those characterized by impaction, comminution, and void creation, present reconstructive challenges distinct from diaphyseal defects. Tibial plateau fractures, distal radius fractures, and calcaneal fractures frequently result in trabecular bone loss and articular surface depression requiring bone grafting to restore anatomy and prevent posttraumatic arthritis [1]. Current standard practice involves autograft, allograft, or synthetic bone substitutes like calcium phosphate cements or granules to fill metaphyseal voids created by fracture reduction and elevation.

Recent advancements in bioprinted scaffolds present notable advantages over conventional bone substitutes for metaphyseal reconstruction. These scaffolds can be fabricated to precisely match the complex geometry of fracture voids, and their controlled pore architecture is optimized to support trabecular bone ingrowth while providing immediate structural reinforcement [11,12]. In the context of tibial plateau fractures, CT-based virtual planning identifies defect volumes following fracture reduction, enabling the design of custom scaffolds that fill voids completely and support subchondral bone restoration.

Biomechanical studies utilizing cadaveric fracture models have demonstrated that bioprinted scaffolds can restore stiffness and resist axial loads at levels comparable to autograft reconstruction, while also eliminating the morbidity associated with graft harvest [37]. Specifically, scaffolds with hexagonal pore configurations exhibit compressive strength approaching that of cortical bone, alongside elastic moduli similar to trabecular bone. This is attributed to the increased contact area between scaffold strands, resulting in an anisotropic structure that enhances load transfer [38].

Distal radius fractures represent another potential application, particularly for elderly patients with osteoporotic bone, where volar locking plates alone may not prevent settling. Bioprinted scaffolds designed to support the subchondral surface while allowing screw passage can be placed through volar or dorsal approaches, providing immediate structural support that prevents articular step-off. The radial metaphyseal region's excellent vascular supply and predominantly cancellous bone composition create favorable conditions for bioprinted scaffold integration and remodeling [39]. Collectively, bioprinted scaffolds represent a significant advancement in managing complex metaphyseal fractures, enabling precise anatomical restoration and improved outcomes through personalized, structurally competent biomaterials that can be designed for compatibility with standard internal fixation systems, including screw trajectories and plate contours.

Fracture Non-unions and Infected Bone Defects

Fracture healing complications such as malunion, nonunion, and infected bone defects remain among the most difficult challenges in orthopaedic surgery. Management is often protracted for established atrophic nonunions with significant bone loss, and prior failed reconstructions often require multiple revision procedures. Conventional strategies revolve around hardware exchange, thorough debridement of nonviable tissue and sclerotic bone, rigid stabilization, and bone grafting. When the osseous defect is extensive, reconstructive options become limited to vascularized bone transfer, bone transport, or complex reconstructions requiring autograft harvest from multiple donor sites [4]. 

Emerging bioprinting technologies offer potential advancements for nonunion repair. Bioprinted scaffolds, especially those incorporating osteoinductive growth factors, may promote union without the morbidity of autograft harvest [16]. The ability to control the release kinetics of factors such as BMP via bioprinting may improve efficacy and limit complications compared to existing carriers like collagen sponges. Advances in scaffold mechanics, driven by synthetic polymers, have yielded bioprinted constructs with elastic moduli suitable for clinical application [40]. 

Infected nonunions represent additional complexity, requiring eradication of infection before any definitive reconstruction [23]. Bioprinted scaffolds loaded with antibiotics have the potential to serve as both local antibiotic delivery systems and bone graft substitutes. Preclinical data with gentamicin- and vancomycin-loaded scaffolds demonstrate sustained local antibiotic release over several weeks, achieving tissue concentrations above the minimum inhibitory concentration for common pathogens while supporting bone regeneration [41]. 

Combat and Blast-Related Extremity Trauma

Military-related extremity trauma, particularly from improvised explosive devices or blast injuries, frequently results in massive bone loss, severe soft tissue compromise, contamination, and vascular injury [1]. These injuries require multiple debridements, staged reconstruction, and prolonged courses of treatment, with high rates of complications such as infection and nonunion. Bioprinted scaffolds designed for such scenarios incorporate antimicrobial agents for infection prevention and pro-angiogenic factors to enhance revascularization, with studies reporting up to a 1.5- to two-fold increase in vascular density and mechanical properties that have demonstrated 20% to 30% reductions in healing time compared to standard scaffold controls [42].

Military medical research organizations have developed acellular, device-based bioprinting systems, both portable and centralized, capable of generating custom polymer-ceramic scaffolds from imaging data within 24 to 48 hours. These remain in preclinical or compassionate use stages, following medical device regulatory pathways, and are not yet approved for bioprinting with living cells. Preclinical models using VEGF and BMP-2 have demonstrated accelerated bone healing and increased vascular density compared to standard-of-care treatments, supporting ongoing research into bioprinting strategies for military trauma [2].

Challenges and future directions

Mechanical Performance and Vascularization Challenges

Despite significant progress in biomaterial development, achieving the mechanical properties of natural cortical bone while preserving the necessary porosity for biological integration remains a significant scientific challenge [19]. Current bioprinted scaffolds typically achieve compressive strengths suitable for metaphyseal applications with supplemental fixation but inadequate for unsupported load-bearing in diaphyseal femur or tibia [17]. Recent innovations in design, such as patient-specific topology optimization, have enabled the development of scaffold architectures with superior strength-to-weight ratios compared to traditional, uniform structures. These approaches leverage individualized loading data to maximize mechanical efficiency, a meaningful step towards clinical viability [9,36]

Post-printing treatments, including thermal annealing, high-pressure compression, or mineralization in simulated body fluid, can substantially enhance mechanical properties [20]. For instance, mineralization processes that deposit calcium phosphate throughout the scaffold’s porous matrix can substantially increase both compressive strength and stiffness while simultaneously improving bioactivity. While such post-processing techniques can yield dramatic improvements, they also introduce additional complexity and increase manufacturing time. Standardization and rigorous validation of these processes are essential to ensure reproducible quality for clinical application [17].

Despite these advancements, vascularization remains the most significant barrier to clinical translation, particularly for large segmental bone defects exceeding 5 cm to 6 cm [39]. Native bone tissue receives an extensive vascular supply, with no osteocyte located more than 100 µm to 200 µm from a capillary [43]. In contrast, bioprinted constructs greater than 2 cm to 3 cm in diameter often develop hypoxic core regions, which severely restrict bone formation. Current research is exploring multiple strategies to address this issue, including the incorporation of angiogenic growth factors (e.g., VEGF), the bioprinting of perfusable channel networks, and coaxial bioprinting to create internal tubular structures that facilitate vascular ingrowth [2,42].

Clinical Integration and Regulatory Pathways

Successful clinical adoption of bioprinted bone grafts requires integration with existing trauma protocols, fixation systems, and surgical workflows rather than wholesale replacement of established practices [17]. Bioprinted scaffolds must be designed to interface with standard internal fixation hardware and complement rather than replace autografts when surgeons deem supplementary grafting necessary. Design features facilitating integration include standardized dimensions compatible with common intramedullary nail diameters, threaded inserts positioned for standard locking screw trajectories, and contours matching plate surfaces for secure fixation [11].

Navigation of regulatory approval processes represents a critical hurdle for clinical translation. Successful clinical adoption of bioprinted bone grafts requires adherence to established regulatory frameworks governing additive manufacturing and patient-specific medical devices. Both the U.S. Food and Drug Administration (FDA) and the European Medicines Agency (EMA) have issued guidance outlining quality assurance, material validation, and process control requirements for 3D-printed medical devices [44, 45]. These emphasize validation of the manufacturing workflow rather than individual patient designs, ensuring reproducibility and biocompatibility across customized implants.

For acellular scaffolds, regulation typically follows the medical device pathway, whereas constructs incorporating cells or growth factors are classified as combination products or advanced therapy medicinal products (ATMPs), requiring additional biological safety and sterility data. These frameworks underscore the need for standardized testing, traceable manufacturing processes, and post-market surveillance before bioprinted implants can achieve full clinical approval. Bioprinted bone grafts potentially fall under multiple regulatory categories depending on composition, and the patient-specific, customized nature creates additional complexity [23]. Regulatory agencies have issued guidance establishing frameworks for validating manufacturing processes rather than individual devices for patient-specific products, requiring demonstration that manufacturing methods consistently produce devices meeting specifications across the range of patient-specific geometries.

Commercial viability depends on reimbursement pathways and health economic value propositions compared to existing treatments [1]. Comparative effectiveness studies with appropriate control groups and sufficient follow-up duration will be necessary to establish value propositions compelling to payers and healthcare systems.

Emerging Technology and Future Directions

Gradient scaffolds represent a significant advancement in the effort to replicate the complex heterogeneous nature of native bone tissue. In vivo, bone exhibits gradual shifts in composition, porosity, and mechanical strength from dense cortical bone to the more porous trabecular regions, a feature critical for its function [25]. With the advent of multi-material bioprinting, researchers can now enable the fabrication of scaffolds with spatially controlled gradients in mineral content, pore size, and growth factor concentration [17]. Such scaffolds are particularly promising for osteochondral defects encountered in periarticular trauma, as they can provide the necessary subchondral bone support while seamlessly transitioning to more porous regions that facilitate cartilage regeneration at the articular surface [16].

Four-dimensional (4D) bioprinting extends traditional 3D printing by incorporating stimuli-responsive materials: those capable of changing their shape, properties, or function in response to environmental triggers such as temperature, pH, or mechanical loading [21]. In trauma medicine, this technology opens the door to scaffolds that can change their configuration after implantation, enabling them to conform to irregular defect geometries or dynamically adapt their mechanical properties in response to physiological loading.

Shape-memory polymers represent one class of smart materials applicable to orthopaedic trauma. These materials can be printed in a temporary configuration conducive to minimally invasive delivery, then activated at body temperature to expand and fill complex bone defects [23]. This strategy has the potential to facilitate percutaneous delivery of large, anatomically matched bone grafts through small incisions, minimizing surgical trauma and expediting patient recovery.

Self-healing materials represent another promising frontier in 4D bioprinting. These scaffolds can autorepair microdamage through reversible chemical bonds or via encapsulated healing agents. For load-bearing trauma applications, self-healing scaffolds could maintain mechanical integrity despite cyclic loading-induced microcracks, extending scaffold lifespan and reducing failure risk during the healing period. Additionally, the development of bone organoids composed of osteoblasts, osteoclasts, endothelial cells, and mesenchymal stem cells offers the possibility of autonomous tissue maturation and vascularization, potentially accelerating integration with host tissue [21]. Collectively, these innovations signal a paradigm shift in the design and function of scaffolds for orthopaedic trauma applications.

Limitations

This narrative review is limited by the heterogeneity of available evidence, much of which is derived from preclinical or benchtop models rather than large human trials. Direct comparisons between studies are constrained by variations in scaffold composition, defect models, and outcome measures. Quantitative synthesis was not feasible due to the absence of standardized reporting on mechanical strength, vascularization outcomes, and clinical endpoints. Furthermore, translational data on regulatory approval, long-term performance, and cost-effectiveness of bioprinted constructs in orthopaedic trauma remain scarce.

Future research recommendations

Future work should prioritize well-designed large-animal studies and early-phase human trials assessing mechanical performance, biological integration, and infection control under load-bearing conditions. Research should focus on optimizing vascularization strategies, including perfusable channel networks and sequential angiogenic-osteogenic factor delivery, and validating in situ and portable bioprinting platforms for intraoperative use. Development of standardized testing protocols and regulatory frameworks for acellular versus bioactive scaffolds is essential to enable safe clinical translation and reproducibility across centers.

## Conclusions

Three-dimensional bioprinting represents a promising advancement in the management of complex bone loss in orthopaedic trauma. Its ability to fabricate patient-specific scaffolds with controlled architecture, mechanical properties, and osteoconductive potential offers an emerging alternative to conventional reconstruction methods such as autografts, allografts, and synthetic bone substitutes. However, current evidence remains largely preclinical, and further clinical validation is required before widespread adoption. Continued innovations in scaffold design, biomaterials, and preclinical research highlight its potential to address the limitations of conventional grafting.

Despite these promising developments, several fundamental challenges must be addressed before widespread clinical implementation. Critical barriers include optimizing mechanical strength for load-bearing use, ensuring vascularization of large constructs, preventing infection in contaminated wounds, and navigating regulatory pathways. Near-term translation will likely begin with smaller defects and low-load regions before progressing to complex reconstructions. The successful integration of bioprinted constructs into existing surgical workflows will necessitate compatibility with standard internal fixation systems and the generation of human-based randomized control trials demonstrating clinical superiority over current gold standard treatments. For patients with severe skeletal injuries, 3D bioprinting holds the promise of faster recovery, fewer complications, and better functional outcomes. Continued multidisciplinary collaboration among engineers, materials scientists, biologists, and orthopaedic surgeons will be essential to realize the full clinical potential of this transformative technology.

## References

[1] Loi F, Córdova LA, Pajarinen J, Lin TH, Yao Z, Goodman SB (2016). Inflammation, fracture and bone repair. Bone.

[2] Park JY, Choi JC, Shim JH (2014). A comparative study on collagen type I and hyaluronic acid dependent cell behavior for osteochondral tissue bioprinting. Biofabrication.

[3] Schmitz JP, Hollinger JO (1986). The critical size defect as an experimental model for craniomandibulofacial nonunions. Clin Orthop Relat Res.

[4] Dimitriou R, Jones E, McGonagle D, Giannoudis PV (2011). Bone regeneration: current concepts and future directions. BMC Med.

[5] Archunan MW, Petronis S (2021). Bone grafts in trauma and orthopaedics. Cureus.

[6] Masquelet AC, Begue T (2010). The concept of induced membrane for reconstruction of long bone defects. Orthop Clin North Am.

[7] Younger EM, Chapman MW (1989). Morbidity at bone graft donor sites. J Orthop Trauma.

[8] Giannoudis PV, Faour O, Goff T, Kanakaris N, Dimitriou R (2011). Masquelet technique for the treatment of bone defects: tips-tricks and future directions. Injury.

[9] Wang W, Yeung KW (2017). Bone grafts and biomaterials substitutes for bone defect repair: a review. Bioact Mater.

[10] Murphy SV, Atala A (2014). 3D bioprinting of tissues and organs. Nat Biotechnol.

[11] Gu BK, Choi DJ, Park SJ, Kim MS, Kang CM, Kim CH (2016). 3-dimensional bioprinting for tissue engineering applications. Biomater Res.

[12] Chen XBD (2019). Extrusion Bioprinting of Scaffolds for Tissue Engineering Applications. https://link.springer.com/book/10.1007/978-3-030-03460-3.

[13] Bishop ES, Mostafa S, Pakvasa M (2017). 3-D bioprinting technologies in tissue engineering and regenerative medicine: current and future trends. Genes Dis.

[14] Kumar A, Nune KC, Misra RD (2016). Biological functionality of extracellular matrix-ornamented three-dimensional printed hydroxyapatite scaffolds. J Biomed Mater Res A.

[15] Paxton N, Smolan W, Böck T, Melchels F, Groll J, Jungst T (2017). Proposal to assess printability of bioinks for extrusion-based bioprinting and evaluation of rheological properties governing bioprintability. Biofabrication.

[16] Park JY, Choi YJ, Shim JH, Park JH, Cho DW (2017). Development of a 3D cell printed structure as an alternative to autologs cartilage for auricular reconstruction. J Biomed Mater Res B Appl Biomater.

[17] Turnbull G, Clarke J, Picard F (2018). 3D bioactive composite scaffolds for bone tissue engineering. Bioact Mater.

[18] Melchels FP, Domingos MA, Klein TJ, Malda J, Bartolo PJ, Hutmacher DW (2012). Additive manufacturing of tissues and organs. Prog Polym Sci.

[19] Baino F, Fiorilli S, Vitale-Brovarone C (2016). Bioactive glass-based materials with hierarchical porosity for medical applications: review of recent advances. Acta Biomater.

[20] Roseti L, Parisi V, Petretta M, Cavallo C, Desando G, Bartolotti I, Grigolo B (2017). Scaffolds for bone tissue engineering: state of the art and new perspectives. Mater Sci Eng C Mater Biol Appl.

[21] Daly AC, Prendergast ME, Hughes AJ, Burdick JA (2021). Bioprinting for the biologist. Cell.

[22] Keriquel V, Oliveira H, Rémy M (2017). In situ printing of mesenchymal stromal cells, by laser-assisted bioprinting, for in vivo bone regeneration applications. Sci Rep.

[23] Ashammakhi N, Hasan A, Kaarela O, Byambaa B, Sheikhi A, Gaharwar AK, Khademhosseini A (2019). Advancing frontiers in bone bioprinting. Adv Healthc Mater.

[24] Hutmacher DW, Schantz JT, Lam CX, Tan KC, Lim TC (2007). State of the art and future directions of scaffold-based bone engineering from a biomaterials perspective. J Tissue Eng Regen Med.

[25] Wu S, Liu X, Yeung KW, Liu C, Yang X (2014). Biomimetic porous scaffolds for bone tissue engineering. Mater Sci Eng R Rep.

[26] Bharadwaz A, Jayasuriya AC (2020). Recent trends in the application of widely used natural and synthetic polymer nanocomposites in bone tissue regeneration. Mater Sci Eng C Mater Biol Appl.

[27] Nyberg E, Rindone A, Dorafshar A, Grayson WL (2017). Comparison of 3D-printed poly-ɛ-caprolactone scaffolds functionalized with tricalcium phosphate, hydroxyapatite, bio-OSS, or decellularized bone matrix. Tissue Eng Part A.

[28] Bose S, Vahabzadeh S, Bandyopadhyay A (2013). Bone tissue engineering using 3D printing. Mater Today.

[29] Kim JY, Ahn G, Kim C (2018). Synergistic effects of beta tri-calcium phosphate and porcine-derived decellularized bone extracellular matrix in 3D-printed polycaprolactone scaffold on bone regeneration. Macromol Biosci.

[30] Khorsandi D, Fahimipour A, Abasian P (2021). 3D and 4D printing in dentistry and maxillofacial surgery: printing techniques, materials, and applications. Acta Biomater.

[31] Bai Y, Yin G, Huang Z, Liao X, Chen X, Yao Y, Pu X (2013). Localized delivery of growth factors for angiogenesis and bone formation in tissue engineering. Int Immunopharmacol.

[32] Bao X, Zhu L, Huang X, Tang D, He D, Shi J, Xu G (2017). 3D biomimetic artificial bone scaffolds with dual-cytokines spatiotemporal delivery for large weight-bearing bone defect repair. Sci Rep.

[33] Jha G, Malasani S, Barakat A, Sola SC, Gera K, Gupta G (2024). Innovative nanotechnological approaches in trauma and orthopaedic surgery: a comprehensive review. Cureus.

[34] Zhang B, Gao L, Ma L, Luo Y, Yang H, Cui Z (2017). 3D bioprinting: a novel avenue for manufacturing tissues and organs. Engineering.

[35] Reichert JC, Cipitria A, Epari DR (2012). A tissue engineering solution for segmental defect regeneration in load-bearing long bones. Sci Transl Med.

[36] Pobloth AM, Checa S, Razi H (2018). Mechanobiologically optimized 3D titanium-mesh scaffolds enhance bone regeneration in critical segmental defects in sheep. Sci Transl Med.

[37] Domingos M, Intranuovo F, Russo T (2013). The first systematic analysis of 3D rapid prototyped poly(ε-caprolactone) scaffolds manufactured through BioCell printing: the effect of pore size and geometry on compressive mechanical behaviour and in vitro hMSC viability. Biofabrication.

[38] Roohani-Esfahani SI, Newman P, Zreiqat H (2016). Design and fabrication of 3D printed scaffolds with a mechanical strength comparable to cortical bone to repair large bone defects. Sci Rep.

[39] Raina DB, Qayoom I, Larsson D (2019). Guided tissue engineering for healing of cancellous and cortical bone using a combination of biomaterial based scaffolding and local bone active molecule delivery. Biomaterials.

[40] Gao C, Deng Y, Feng P (2014). Current progress in bioactive ceramic scaffolds for bone repair and regeneration. Int J Mol Sci.

[41] Zhang Y, Zhai D, Xu M, Yao Q, Zhu H, Chang J, Wu C (2017). 3D-printed bioceramic scaffolds with antibacterial and osteogenic activity. Biofabrication.

[42] Shahabipour F, Ashammakhi N, Oskuee RK, Bonakdar S, Hoffman T, Shokrgozar MA, Khademhosseini A (2020). Key components of engineering vascularized 3-dimensional bioprinted bone constructs. Transl Res.

[43] Hadjidakis DJ, Androulakis II (2006). Bone remodeling. Ann N Y Acad Sci.

[44] (2021). Technical considerations for additive manufactured medical devices | FDA. https://www.fda.gov/regulatory-information/search-fda-guidance-documents/technical-considerations-additive-manufactured-medical-devices.

[45] (2025). Guideline on quality, non-clinical and clinical requirements for investigational advanced therapy medicinal products in clinical trials - scientific guideline | European Medicines Agency (EMA). https://www.ema.europa.eu/en/guideline-quality-non-clinical-clinical-requirements-investigational-advanced-therapy-medicinal-products-clinical-trials-scientific-guideline.

